# It is not only fluids: the impact of hydration protocols used for the prevention of contrast nephropathy on renal oxygenation

**DOI:** 10.1080/0886022X.2025.2528889

**Published:** 2025-07-09

**Authors:** Samuel N. Heyman, Richard Solomon, Zaid Abassi

**Affiliations:** ^a^ Department of Medicine, Hadassah Hebrew University Hospital, Jerusalem, Israel; ^b^ Department of Nephrology, The University of Vermont Medical Center, Burlington, Vermont, USA; ^c^ Department of Physiology, Bruce Rappaport School of Medicine, Technion, Haifa, Israel; ^d^ Department of Laboratory Medicine, Rambam Health Care Campus, Haifa, Israel

**Keywords:** Radiocontrast nephropathy, hypoxia, fluids, diuretics, furosemide, medulla

## Abstract

Fluid administration is the mainstay intervention effective in the prevention of radiocontrast-associated nephropathy (CAN) in high-risk patients. Vigorous hydration shortens intratubular solute transit-time and reduces tubular intraluminal concentration of contrast media (CM), decreasing exposure of tubular cells to CM and reducing renal parenchymal retention of the nephrotoxin. Lowered plasma and urine viscosity might also improve vasa recta flow and renal interstitial pressure, improving compromised renal parenchymal microcirculation and oxygenation. Herein we emphasize the overlooked plausible role of down-regulation of tubular transport, generated by vigorous hydration in the mitigation of medullary hypoxia and hypoxic medullary damage generated in CAN. Volume expansion triggers natriuretic peptides that improve renal parenchymal oxygenation and may attenuate hypoxic renal injury. Furthermore, enhanced large-volume hydration protocols used for high-risk patients undergoing coronary interventions or transcatheter aortic valve implantation include the administration of furosemide. Loop diuretics block oxygen consumption in medullary thick ascending limbs, improve medullary oxygenation and prevent outer medullary injury in experimental CAN. Thus, fluids are likely not the sole issue, and restoration of medullary oxygenation is critical in attenuating the risk of CAN by large volume hydration protocols for high-risk patients.

## Introduction

The administration of iodinated radiocontrast material (CM) may lead to radiocontrast-associated nephropathy (CAN) [1–3]. Renal parenchymal hypoxia and consequent formation of reactive oxygen species play a central role in the pathogenesis of CAN, reflecting reduced renal blood flow and oxygen supply, concomitantly with enhanced oxygen needs for tubular transport workload. These changes in oxygen supply and demand are especially detrimental in the outer medulla, characterized by limited regional blood supply and intense tubular transport activity, with consequent low pO_2_ under normal conditions [4,5]. Preexisting chronic kidney disease (CKD) and diabetes are the principal risk factors predisposing to CAN [6], along with hemodynamic compromise during cardiac interventions and the volume of CM injected. Among additional risk factors are advanced age, anemia, and additional co-morbidities [2,7]. Noteworthy, most of these risk factors predispose to the intensification of medullary hypoxia, with altered mechanisms that maintain medullary oxygenation by matching regional oxygen supply and expenditure [2,4].

The expanding usage of CM for contrast-enhanced imaging and for intravascular interventions is the reason why CM remains a leading cause of iatrogenic acute kidney injury (AKI) [1,3]. Yet, despite patients’ risk stratification and selection, the development of safer low- or iso -osmolar agents, and the strive to avoid large volumes of administered CM, CAN remains a nagging problem in high-risk patients, with some 0.3%-0.5% of patients requiring dialysis and others revealing increased mortality and morbidity and rising odds for CKD progression [3,8,9]. For instance, as opposed to a widespread belief that CAN following a standard contrast-enhanced CT is a myth [9], it is not the case in patients with advanced renal failure, despite the small volumes of CM required for such procedures. In fact, iodine-based contrast enhancement increased the likelihood of post-CT AKI in hospitalized patients by 51%, as compared to patients undergoing non-enhanced imaging (confidence interval, 1.23-2.05) [10]. The risk of CAN is especially prominent in patients undergoing intravascular interventions that require larger volumes of CM, as opposed to those used for contrast-enhanced CT. Furthermore, these patients often have CKD and diabetes and are prone to hemodynamic instability and reduced renal perfusion [7]. The incidence of CAN may exceed 30% in such patients with baseline creatinine > 2 mg/dL [2] and is higher in emergent procedures characterized by hemodynamic instability (12% in ST-elevation MI, vs.9.2% in patients with non-ST-elevation MI and 4.5%, only, in patients undergoing elective coronary interventions) [11].

## Hydration for the prevention of AKI in general

The administration of fluids is the cornerstone of preventive measures against CAN, irrespective to its composition and mode of administration [12,13]. The occurrence of CAN following coronary intervention declined roughly by 50% with hydration protocols, as compared with non-hydrated control groups [14,15]. The renoprotective impact of hydration seems to be true also in other scenarios leading to AKI, such as rhabdomyolysis [16] or ischemic acute tubular necrosis (ATN) following cardiac surgery [17]. In the same fashion, fluids plausibly attenuate the risk of drug-induced AKI such as cyclosporine nephropathy post kidney transplantation, associated with intense renal vasoconstriction [18] or nephrotoxic- or crystal-induced AKI, such as aminoglycoside [19] and acyclovir nephropathy [20], reflectively.

Consensus exists regarding mechanisms involved in the attenuation of renal injury with fluids in AKI in general. First, it eliminates any possible component of pre-renal failure by improving the patient’s volume status. Furthermore, hydration leads to reduced tubular intraluminal concentration of the nephrotoxic agent and shorten urine transit time, reducing tubular uptake in a nephrotoxin-specific tubular cell type. Enhanced tubular intraluminal urine flow also prevents tubular obstruction by detached and fragmented tubular cells and cellular casts in ischemic or nephrotoxic ATN, by crystals, for instance in acyclovir nephropathy, or by diverse intraluminal precipitations of various compounds, such as heme pigments, Tamm Horsfall protein, filtered paraproteins etc.

Accordingly, guidelines recommend intravascular volume expansion also for CAN prophylaxis, yet there is no consensus regarding the optimal hydration regime [13,21]. The dilemma is to achieve sufficient hydration status in high-risk patients, especially those administered with large volumes of contrast media during cardiovascular interventions, without increasing the risk of pulmonary congestion. Two tailored hydration regimens have been suggested and thoroughly investigated: 1- left ventricular end-diastolic pressure (LVEDP)-guided hydration and 2- urine flow rate-guided hydration using the RenalGuard system [12] or using a non-automated adjustment [22]. Protocols of forced hydration, detailed elsewhere [12,13], include a large volume load followed by a continuous drip of fluids, coupled with the administration of furosemide, matching drip infusion to urine output. Irrespective to the fluid regimen chosen, large urine volume (above 150 mL/h and aimed at 300 mL/h) seems to be the goal to minimize the risk of CAN in high-risk patients undergoing cardiovascular interventions [12,13].

## Mechanisms by which hydration directly attenuates CAN

CM hardly penetrate the renal tubular epithelium, though very small amounts apparently reach the interstitium and generate inflammation and toxicity [23]. Vigorous hydration might further reduce renal parenchymal uptake of CM, thanks to lowering intraluminal concentration of the contrast agent and to a shortened tubular intraluminal transit time. Hydration might also decrease the exposure of tubular cells to high concentrations of CM, attenuating a possible direct plain cytotoxic injury, noted in experimental settings *in vitro* only [24] but not morphologically *in vivo* [25,26]. Indeed, the incidence of CAN and biomarkers of kidney injury are directly related to the density of nephrograms obtained immediately post the cardiac procedure [27]. Enhanced matched diuresis is known to reduce the density of nephrograms post contrast exposure [28]. This supports the idea that dilution of contrast and wash-out from the nephron may be important for the beneficial effects of high urine outputs. However, this would not easily explain the benefit of balanced forced hydration on post-operative AKI reported in patients undergoing CABG at the absence of CM [17], unless another toxin such as free hemoglobin is a major mechanism of injury [29].

Dilution of urine and plasma by hydration protocols may also attenuate the risk of CAN by reducing urine and plasma viscosity. This mechanism might be principally important while using iso-osmolar agents, characterized by high viscidness [30]. Reducing viscosity by fluid administration could facilitate urine flow and vasa recta blood flow, decrease renal parenchymal interstitial pressure and improve renal microcirculation [30,31]. Yet, as outlined elsewhere in detail [4,5], since renal medullary hypoxic injury with the consequent formation of reactive oxygen species play a central role in CAN, it is tempting to assume that the kidney protection with hydration may be driven principally through improved renal oxygenation balance.

## The impact of CM on renal medullary oxygenation

Medullary pO2 is normally below 30 mmHg, reflecting intense oxygen demand for tubular transport, at a region with a limited oxygen supply, delivered by vasa recta [32]. Medullary oxygenation balance is maintained by mechanisms matching regional blood flow and oxygen expenditure. Nitric oxide, vasodilating prostaglandins (PGE2, PGI2), and adenosine are principal protective agents in sustaining medullary oxygen sufficiency, by enhancement of regional oxygen supply and suppression of tubular transport activity [33]. CM intensify physiologic medullary hypoxia (as evidenced by oxygen microelectrodes, by blood-oxygen level-dependent magnetic resonance imaging [BOLD MRI] and by immunostaining for pimonidazole adducts and for hypoxia-inducible factors), likely through enhancement of distal tubular transport load, combined with compromised medullary blood flow [4]. Plausibly, the higher risk of CAN following the use of the old generation high-osmolar ionic agents is associated with osmotic diuresis caused by these agents, that intensifies solute delivery to the distal nephron. Interestingly, renal protective physiological mechanisms that maintain medullary oxygen sufficiency are defective in medical conditions predisposing to CAN, such as CKD, aging or diabetes [34,35], underscoring the role of hypoxic damage in AKI following radiocontrast studies or interventions.

## The impact of hydration protocols on renal medullary oxygenation

Hydration could improve medullary oxygenation following the administration of CM by enhancement of medullary blood flow and plausibly by suppression of tubular transport, mediated by endogenous natriuretic peptides and the generation of prostaglandin PGE2 and nitric oxide. Natriuretic peptides relax the renal microvasculature, and vasa recta express its receptors [36]. Natriuretic peptides also reduce oxygen expenditure for tubular transport along the nephron [37] and could mitigate hypoxic stress invoked by CM. Furthermore, renal parenchymal expression of corin [38] and perhaps furin, might permeate endogenous renal generation of natriuretic peptides in response to hydration, and may reduce the risk of CAN through the attenuation of oxygen expenditure for tubular transport. Noteworthy, high-osmolar CM themselves enhance the generation of natriuretic peptides [39], likely through an abrupt rise in intravascular volume, and may, to some extent, counteract renal vasoconstriction triggered by various endogenous systems, including CM-induced endothelin release [39,40], triggered by evolving renal hypoxia [41]. Natriuretic peptides were found to attenuate ischemic AKI following cardiac surgery in some studies [42,43], but the renoprotective effect was not shown in other comparable clinical trials [44,45], highlighting limitations related to small cohorts of patients [46]. Atrial natriuretic peptide (ANP) was also reported to attenuate CAN following coronary interventions [47].

As to the impact of hydration on augmenting vasa recta blood flow and medullary oxygen supply, this response is unfortunately hampered in aged or diabetic patients with altered nitrovasodilation and defective prostaglandin synthesis, and renal medullary oxygenation might even decline, due to enhanced solute delivery for tubular transport in the distal nephron [48].

Therefore, we would like to emphasize that other than improving oxygen supply, attenuation of regional oxygen expenditure for tubular transport might be an additional pivotal mechanism in the prevention of CM-associated medullary hypoxic damage by hydration protocols [4]. First, diuresis down-regulates tubular transport by the generation of PGE2 and nitric oxide, as well as through increasing levels of natriuretic peptides, mentioned above, with the potential prevention of medullary hypoxic damage.

## The impact of adding loop diuretics on renal medullary oxygenation

Protocols of enhanced diuresis in patients undergoing coronary interventions or trans-catheter aortic valve implantation (TAVI) include the administration of loop diuretics. The addition of furosemide facilitates the administration of large volumes of fluids to high-risk patients with cardiac dysfunction, prone to pulmonary congestion [49], with minute to minute careful matching of urine output and fluid load to eliminate effective volume depletion or to prevent volume overload precipitating heart failure. As detailed elsewhere regarding administered fluids and furosemide dosage [12,13], such protocols of enhanced balanced diuresis combined with furosemide were consistently highly effective in reducing the risk of CAN in high-risk individuals. A meta-analysis of such studies revealed a significant reduction in the incidence of CAN in both settings, as compared with regular hydration protocols (6.7% vs. 15.7%; 95% CI: 0.27 to 0.54 for coronary interventions, and 15.6% vs. 26.9%; 95% CI: 0.35 to 0.82, for TAVI, respectively) [50]. The same principle of forced diuresis combined with furosemide with non-automated adjustment of fluid intake and output led to a comparable reduction in the occurrence of CAN in patients with CKD, 8% vs. 14% in controls [22].

**Figure 1.**
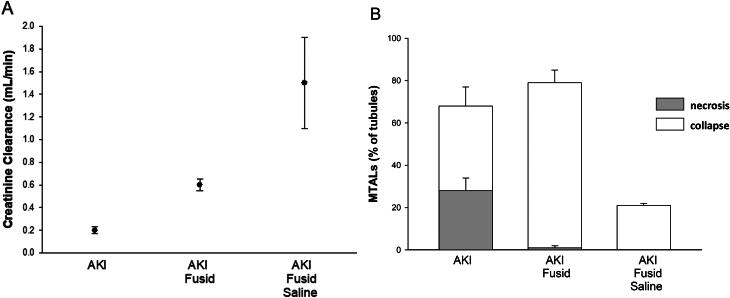
The effect of furosemide alone and of furosemide, combined with hydration on A) creatinine clearance and B) renal morphology in rats subjected to a model of contrast-associated nephropathy (CAN). Furosemide alone abolished medullary thick lamb (mTAL) necrosis by 24 h (gray bars). However, renal dysfunction was hardly affected. This likely reflects dehydration, manifested by a doubling of collapsed mTALs, illustrated as white bars (conceivably due to reduced single nephron glomerular filtration rate and enhanced solute reabsorption in proximal tubules). By contrast, kidney function remained preserved when furosemide was complemented by hydration, eliminating dehydration and pre-renal failure, as evidenced by a parallel reduction of the extent of collapsed tubules. (adopted from [55]). data is presented as means ± SD.

Furosemide blocks NaKCl2 co-transport in medullary thick limbs (mTALs) and markedly improve medullary oxygenation through the cessation of oxygen consumption for tubular transport, even though regional blood flow declines [51]. Medullary oxygenation, determined non-invasively by BOLD MRI substantially increases in individuals following the administration of furosemide, though for obscure reason this response is blunted in older patients [52]. In this perspective, furosemide was found to attenuate hypoxic damage in mTALs in isolated perfused kidneys [53] and even prevents ischemic injury in neighboring S3-segments of proximal tubules, likely reflecting improved ambient oxygenation affecting both tubule types [54]. In the same manner, furosemide was found to reverse CM-related intensified medullary hypoxia [26] and has prevented regional hypoxic mTAL damage in the outer medulla in an in-vivo rat model of CAN [55]. Interestingly, as illustrated in Figure 1, adopted from this study, substantial attenuation of tubular hypoxic damage with furosemide alone in this model was not associated with significantly improved kidney function, despite the mitigation of tubular damage, reflecting a pre-renal component of kidney failure (manifested by extensive mTAL collapse). By contrast, furosemide combined with hydration resulted in the elimination of tubular collapse, the morphologic evidence of dehydration, and led to preserved kidney function. These findings likely explain the failure of furosemide to prevent CAN in two subsequent clinical trials we conducted in the early nineties [56,57]. In fact, furosemide even worsened kidney function in patients undergoing angiographies in these studies, likely caused by insufficient hydration with evolving pre-renal failure, as evidenced by weight reduction in patients administered with furosemide [56]. A parallel dichotomized situation was likely detected by careful statistical analysis in the broader perspectives of AKI in general, where plausibly pre-renal failure in acutely ill hospitalized patients could be attributed to the use of loop diuretics [58], whereas the same treatment predicted a better renal outcome in patients hospitalized with AKI [59]. To conclude, there is a sound physiologic basis for including loop diuretics in protocols of CAN prevention in high-risk patients, in order to attenuate transport workload and consequent medullary hypoxia, providing vigorous hydration is maintained to conserve the hydration status. Yet, it should be emphasized that, for ethical reasons, the independent protective effect of furosemide against CAN, other than the proved reversal of medullary hypoxia, cannot be evaluated in clinical trials.

## Summary and conclusions

**Figure 2.**
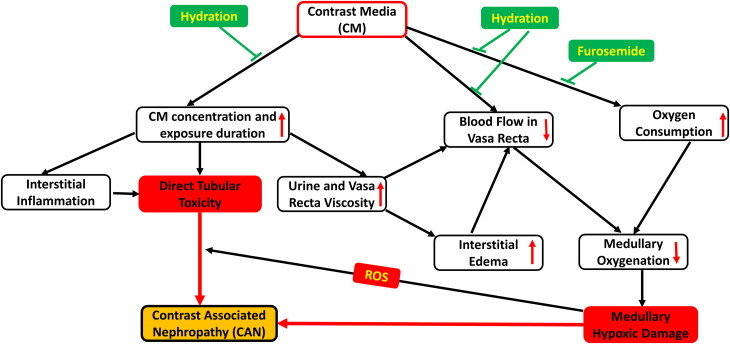
A scheme illustrating pathways generating contrast-associated nephropathy (CAN) and the mechanisms by which hydration and furosemide are believed to be renoprotective.

Apparently, as illustrated in Figure 2, fluids per se are renoprotective in a host of clinical setups, including CAN, through urinary dilution and swift elimination of nephrotoxins, attenuating its renal parenchymal uptake and direct nephrotoxicity. Additionally, hydration may improve renal medullary oxygenation, in part through the attenuation of oxygen utilization for tubular transport. Animal studies and clinical trials indicate that further reduction of tubular transport pharmacologically may provide an additional renal protection in the case of CAN by reversal of critical medullary hypoxia. Thus, the clinically effective combination of vigorous balanced hydration and loop diuretics in high-risk patients is a physiologically sound approach in the prevention of CAN, with concomitant reduction of renal retention and exposure to high concentrations of CM and the attenuation of medullary hypoxia *via* both improved microcirculation and suppressed regional oxygen expenditure. The combination of forced hydration and furosemide during radiocontrast studies and interventions is particularly important in patients with severely impaired cardiac performance prone to pulmonary congestion, and requires close monitoring of hydration status and hemodynamic stability, The physiologic rationale of this approach, already validated by clinical outcomes, might be confirmed in the future by assessing the impact of hydration and diuretics on renal oxygenation and by the determination of biomarkers of renal injury in high-risk patients following expose to CM, Finally, since CAN is associated with increased risk of CKD progression, it is suggested to explore if the implementation of effective strategies that mitigate medullary hypoxia and the risk to develop CAN also lead to better long-term renal outcomes in high-risk patients subjected to imaging or interventional procedures with CM.

## Data Availability

Data may be made available to qualified researchers for approved scientific uses.
